# Modeling Human Severe Combined Immunodeficiency and Correction by CRISPR/Cas9-Enhanced Gene Targeting

**DOI:** 10.1016/j.celrep.2015.08.013

**Published:** 2015-08-28

**Authors:** Chia-Wei Chang, Yi-Shin Lai, Erik Westin, Alireza Khodadadi-Jamayran, Kevin M. Pawlik, Lawrence S. Lamb, Frederick D. Goldman, Tim M. Townes

**Affiliations:** 1 Department of Biochemistry and Molecular Genetics; 2 Department of Medicine, Division of Hematology/Oncology; 3 Cell Therapy Lab; 4 Department of Pediatrics, Division of Hematology/Oncology; 5 UAB Stem Cell Institute, Schools of Medicine and Dentistry, University of Alabama at Birmingham, Birmingham, AL 35294, USA

## Abstract

Mutations of the Janus family kinase JAK3 gene cause severe combined immunodeficiency (SCID). JAK3 deficiency in humans is characterized by the absence of circulating T cells and natural killer (NK) cells with normal numbers of poorly functioning B cells (T^−^B^+^NK^−^). Using SCID patient-specific induced pluripotent stem cells (iPSCs) and a T cell in vitro differentiation system, we demonstrate a complete block in early T cell development of JAK3-deficient cells. Correction of the JAK3 mutation by CRISPR/Cas9-enhanced gene targeting restores normal T cell development, including the production of mature T cell populations with a broad T cell receptor (TCR) repertoire. Whole-genome sequencing of corrected cells demonstrates no CRISPR/Cas9 off-target modifications. These studies describe an approach for the study of human lymphopoiesis and provide a foundation for gene correction therapy in humans with immunodeficiencies.

## In Brief

Using SCID patient-specific induced pluripotent stem cells (iPSCs) and a T cell in vitro differentiation system, Chang et al. define a block in early T cell development of JAK3-deficient cells. Correction of the JAK3 mutation by CRISPR/Cas9-enhanced gene targeting restores normal T cell development.

## Graphical Abstract

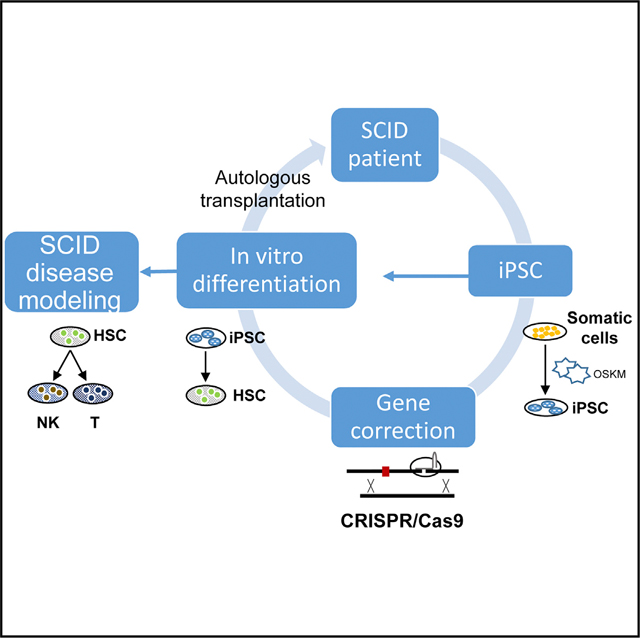

## INTRODUCTION

Severe combined immunodeficiency (SCID) describes patients with severe defects in T cells with or without accompanying defects in B cells. Naturally occurring mutations in the JAK3 gene (autosomal recessive) and the X-linked common gamma chain γ_C_ gene (IL-2RG) are the most common T^−^B^+^ immunodeficiencies. Gene ablation experiments in mice demonstrate that Jak3 is critical for early T cell differentiation and Jak3 knockout mice were found to have severely reduced numbers of mature B cells in the bone marrow and in the periphery (Nosaka et al., 1995; Thomis et al., 1995). The similarity of phenotypes of γc and JAK3 SCID suggests that the primary function of JAK3 is to transduce signals from γc-dependent cytokine receptors (IL-2R, IL-4R, IL-7R, IL-9R, IL-15RA, and IL-21R) to transcription factors (STATs) that activate downstream genes. Mice lacking Jak3 have a profound decrease in thymus cellularity. However, the residual thymocytes proceed to develop into mature T cells and reconstitutetheperipheralpopulation.Incontrast,JAK3-deficientpatients have few, if any, peripheral T cells (Notarangelo et al., 2001; Russell et al., 1995).To date, little is known about how JAK3mutations affect human lymphocyte progenitor development.

Allogeneic hematopoietic stem cell transplantation is currently the only established therapy for SCID; however, delayed immune recovery and graft-versus-host disease present significant risks (Pai et al., 2014). Treatment by retroviral-based gene therapy has been successfully demonstrated for X-linked SCID (Hacein-BeyAbina et al., 2014) and ADA-SCID (Ferrua et al., 2010). However, severe adverse effects of insertional mutagenesis have been observed with retroviral gene therapy (Hacein-Bey-Abina et al., 2003, 2008). Self-inactivating lentiviral vectors have been used effectively in recent clinical trials, but long-term follow-up is needed to thoroughly address safety concerns (Aiuti et al., 2009; Biffi et al., 2013; Sauer et al., 2014). An alternative therapeutic strategy is one in which patient-specific induced pluripotent stem cells (iPSCs) are derived, and disease-causing mutations are corrected by gene targeting (Takahashi et al., 2007; Takahashi and Yamanaka, 2006; Yu et al., 2007). These corrected iPSCs could then be differentiated into hematopoietic progenitors for transplantation into patients to treat the disease (Hanna et al., 2007). The recent development of CRISPR/Cas9-enhanced gene targeting dramatically advances the practicality of this strategy (Cong et al., 2013; Mali et al., 2013).

IPSC technology combined with in vitro differentiation systems also provides a powerful platform to recapitulate in vivo development. We and other groups have shown that OP9 stromal cells transduced with Notch ligand Delta-like-1 or Delta-like-4 (OP9-DL1/4) can contribute to a T cell inductive environment (Chang et al., 2014; Schmitt et al., 2004; Schmitt and Zúñiga-Pflucker, 2002). OP9-DL1/4 cells efficiently induce T lymphopoiesis from iPSC-derived CD34^+^ hematopoietic progenitor cells (HPCs), and these HPCs can be differentiated into functional CD8 T cells (Dervovic et al., 2012). Despite the lack of proper major histocompatibility complex (MHC) expression on OP9 cells, the OP9-DL1/4 system has provided a valuable method to study early T cell commitment and thymocyte maturation in vitro (de Pooter and Zúñiga-Pflucker, 2007).

In this paper, we demonstrate that differentiation of JAK3-deficient human T cells is blocked at an early developmental stage. Similar to previous studies in mouse models, JAK3-deficient early human T cell progenitors undergo apoptosis at a high rate due to low expression of BCL2. We also demonstrate that correction of the human JAK3 mutation by CRISPR/Cas9-enhanced gene targeting restores the differentiation potential of early T cell progenitors. These corrected progenitors are capable of producing NK cells and mature T cell populations expressing a broad repertoire of T cell antigen receptors (TCRs). These studies establish a powerful system for determining the mechanism of immunodeficiency in human SCID patients and for testing pharmacological and genetic therapies for the disorder.

## RESULTS

### JAK3-Deficient Human T Cells Express Low Levels of BCL2 and Die at an Early Developmental Stage

**Figure 1.**
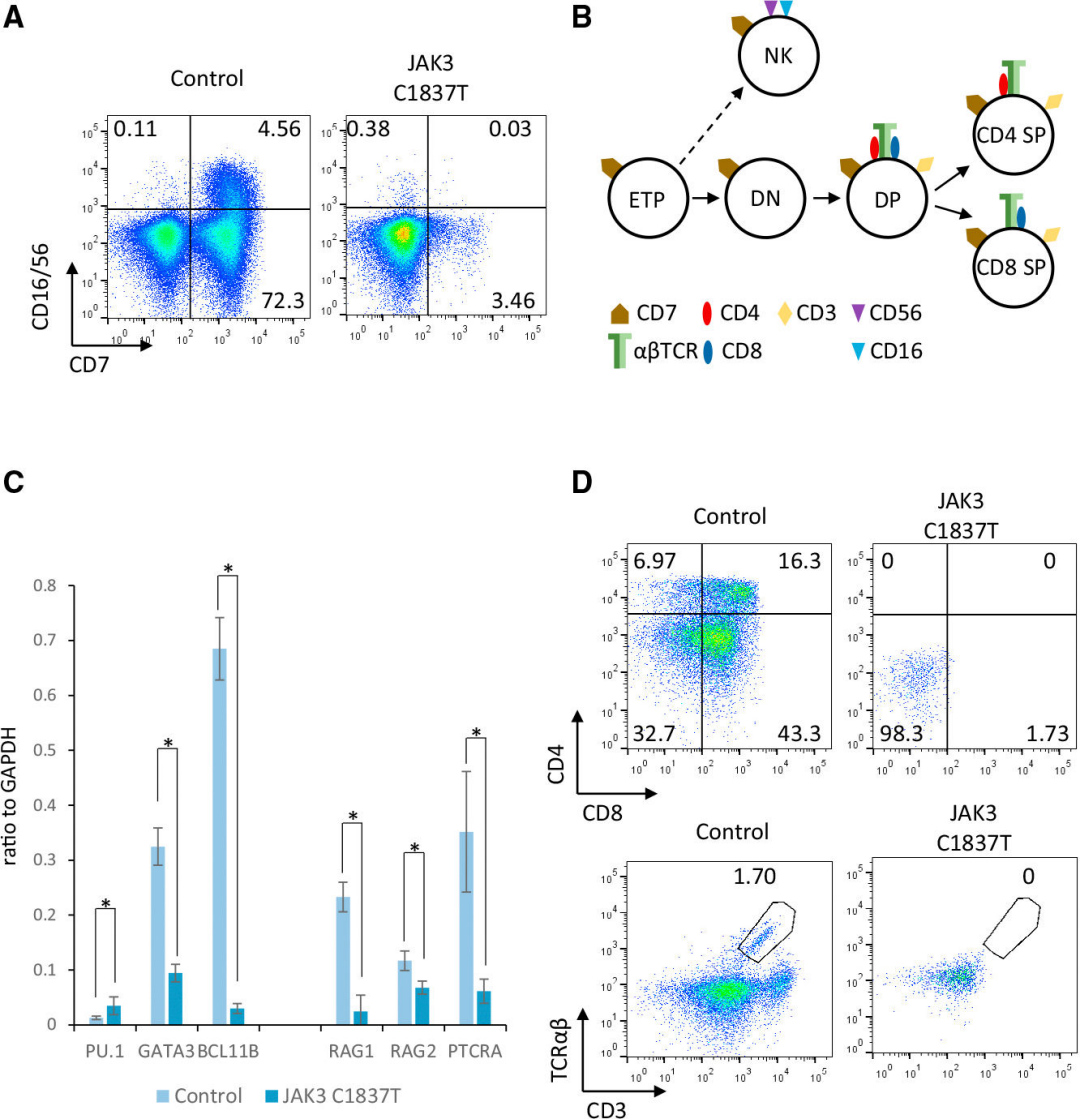
In Vitro Differentiation of JAK3 C1837T Patient iPSCs Recapitulates SCID Phenotypes (A) Flow cytometry of iPSC-derived T cells. JAK3 WT iPSCs (Control) and JAK3-deficient iPSCs (JAK3 C1837T) were differentiated into CD34^+^ cells on OP9 stromal cells and, subsequently, into NK and T cells on OP9-DL4 monolayers. T and NK cell differentiation from JAK3-deficient iPSCs was dramatically decreased compared to controls; only a small population of CD7^+^CD16^−^CD56^−^ T cells or CD7^+^CD16^+^CD56^+^ NK cells was observed at T cell induction day 14. (B) Diagram of early T cell development. In thethymus, early T cell progenitors (ETP) differentiate from CD4–CD8^−^ double-negative (DN) to CD4^+^CD8^+^ double-positive (DP) and, subsequently, to CD4^+^ or CD8^+^ single-positive stages. In an alternative pathway, early T cell progenitors differentiate into NK cells; the dotted line indicates multiple steps in the pathway to mature CD7^+^CD16^+^CD56^+^ NK cells. (C) RT-qPCR assays for transcripts of key genesthat regulate early events during specification of the T cell lineage. RNA levels are shown relative to GAPDH expression. Data are shown as the mean ± SD. *p < 0.05 (t test). (D) Flow cytometry of iPSC-derived T cells. JAK3 WT iPSCs (Control) and JAK3-deficient iPSCs (JAK3 C1837T) were differentiated into CD34^+^ cells on OP9 stromal cells and, subsequently, into T cells on OP9-DL4 monolayers. At T cell induction day 28, no CD4^+^CD8^+^ DP, CD4^+^ SP, or CD8^+^ SP T cells were detected (top), and neither CD3 or TCRαβ were expressed in JAK3-deficient iPSC-derived T cells (JAK3 C1837T) (lower).

IPSCs were generated from skin keratinocytes (Chang et al., 2009) of a SCID patient homozygous for a C > T nucleotide substitution in exon 14 of the JAK3 gene. This mutation replaces a CGA codon (arginine at 613) with a TGA stop codon (p.R613X). The 4-month-old patient presented with a T^−^B^+^NK^−^ clinical phenotype (see Experimental Procedures). To determine whether this SCID phenotype can be recapitulated in vitro, we attempted to differentiate patient-specific iPSCs to T lymphocytes using our previously published two-step OP9 and OP9-DL4 system (Chang et al., 2014). JAK3-deficient iPSCs grew at a rate comparable to control iPSCs derived from healthy donors, and these iPSCs efficiently differentiated into CD34^+^ hematopoietic progenitors (HPs) on OP9 stromal cell monolayers. However, when the JAK3-deficient, iPSC-derived CD34^+^ HPs were plated onto OP9-DL4 stromal monolayers, NK and T cell differentiation was dramatically decreased compared to controls (Figure 1A). Only a small population of CD7^+^CD16^−^CD56^−^ T cells or CD7^+^CD16^+^CD56^+^ NK cells was observed at T cell induction day 14 (Figure 1A).

The transitions of early T cell progenitors (ETPs) from CD4^−^CD8^−^ double-negative (DN) → CD4^+^CD8^+^ double-positive (DP) → CD4^+^ single-positive (SP) or CD8^+^ single-positive (SP) T cells are directed by precise activation and repression of specific transcription factors (Rothenberg et al., 2008) (Figure 1B). In control cells (Figure 1C), silencing of PU.1 gene expression and induction of GATA3 and BCL11B gene expression direct early hematopoietic progenitors to proceed to the onset of T lineage commitment. In JAK3-deficient cells, expression of the PU.1 gene was not completely silenced, induction of the GATA3 and BCL11B genes was significantly lower than controls, and T cell specification was severely limited. However, low-level expression of the T cell-specific genes RAG1, RAG2, and PTCRA suggests that JAK3-deficient cells can progress at low efficiency to an early T cell progenitor stage. Jak3 knockout (KO) mice have a small thymus due to a block in thymocyte differentiation at the CD4^−^CD8^−^ double-negative 2 (DN2) stage prior to productive TCR rearrangement. Interestingly, some residual thymocytes in Jak3 knockout (KO) mice develop into mature T cells and reconstitute the peripheral population (Eynon et al., 1999); this does not occur in human JAK3-deficient patients. To further understand the developmental defects resulting from JAK3 deficiency in humans, we assayed T cell lineage commitment and maturation in JAK3-deficient cells compared to normal JAK3 WT controls. IPSC-derived CD34^+^ cells were plated onto OP9-DL4 monolayers, and cells were harvested and analyzed for lymphocyte markers at T cell induction day 28. No CD4^+^CD8^+^ DP, CD4^+^ SP, or CD8^+^ SP T cells were detected in JAK3-deficient cells. Moreover, CD3 and TCR αβ were not significantly detected in JAK3-deficient cells (Figure 1D). The complete absence of CD4^+^CD8^+^ cells indicates that human JAK3-deficient cells arrest at the CD4^−^CD8^−^ DN stage. Low expression of the DN3-associated genes PTCRA, RAG1, and RAG2 suggests that human JAK3-deficient cells arrest at or before the DN2 stage. Unlike the results in Jak3 KO mice in which residual thymocytes develop past the CD4^−^CD8^−^ DN stage into mature T cells that partially reconstitute the peripheral blood system, our data demonstrate a complete block of T cell development in human JAK3-deficient cells. This block is consistent with the absence of mature T cells in peripheral blood of JAK3-deficient human SCID patients.

**Figure 2.**
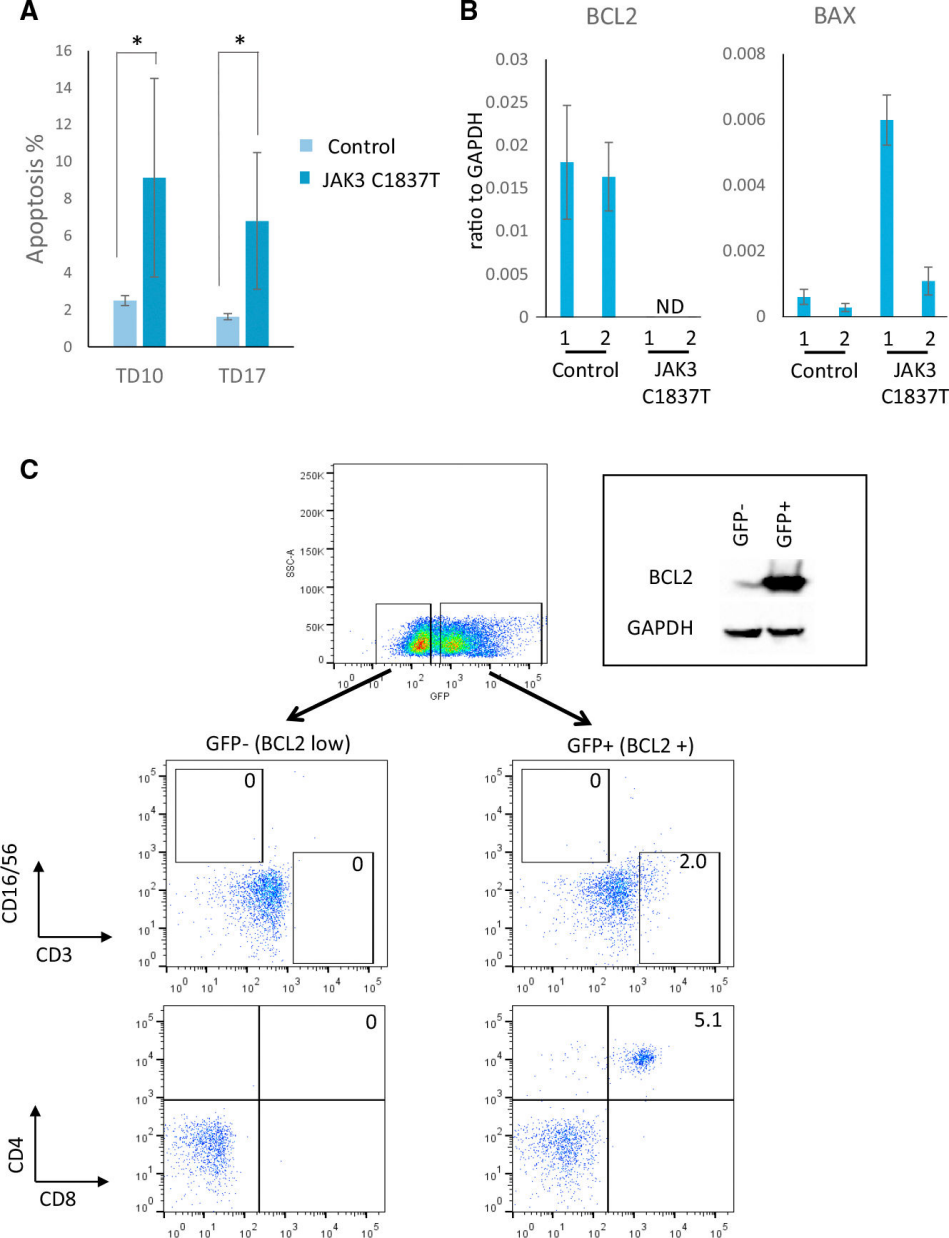
BCL2 Partially Rescues T Cell Developmental Defects in JAK3-Deficient, In-Vitro-Derived Cells (A) Apoptosis of JAK3-deficient, iPSC-derived T cells compared to JAK3 WT controls. Annexin V-positive cells were analyzed at T cell induction day 10 (TD10) and 17 (TD17). Four independent experiments were performed with control JAK3 WT cells (Control, light blue), and five independent experiments were performed with JAK3-deficient cells (JAK3 C1837T, dark blue). Data are shown as the mean ± SD. *p < 0.005 (t test). (B) RT-qPCR assays for anti-apoptotic BCL2 andpro-apoptotic BAX expression in two lines (1 and 2) from JAK3 WT (Control) and JAK3-deficient cells (JAK3 C1837T). ND, not detected. RNA levels are shown relative to GAPDH expression. (C) Flow cytometry of JAK3-deficient iPSC-derivedT cells transduced with BCL2–2A-GFP lentivirus to assess effects on NK (CD16^+^56^+^) and T cell (CD3^+^) development and on CD4^+^CD8^+^ DP to CD4^+^ SP or CD8^+^ SP T cell maturation. (top right of C) Western blot of BCL2 protein in GFP^−^ and GFP^+^ sorted populations.

The profound defects in lymphocyte development of JAK3-deficient cells can be explained by the absence of IL-7 signaling, which plays an important role in lymphoid progenitor survival (Li et al., 2004; von Freeden-Jeffry et al., 1997) and differentiation (Kang et al., 1999). IL-7/JAK3 signaling maintains thymocyte homeostasis by regulating the BCL2 family of apoptotic regulators. Thymocytes and peripheral T cells from Jak3 KO mice have a high apoptotic index in part through selectively elevating Bax, a pro-apoptotic factor, and by reducing expression of Bcl2, an anti-apoptotic factor (Wen et al., 2001). Similarly, we observed an increase in apoptosis of in-vitro-derived human JAK3-deficient cells compared to controls at T cell induction day 10 (9%–2.2%) and T cell induction day 17 (7%–1.9%) (Figure 2A). Consistent with this phenotype, BAX levels were increased and BCL2 levels were reduced in JAK3-deficient cells compared to controls (Figure 2B).

Forced expression of Bcl2 rescues T but not B or NK cell development in γc-deficient mice (Kondo et al., 1997). Transplantation of Jak3 KO mice with Bcl2-expressing Jak3 KO bone marrow cells also improves peripheral T cell numbers (Wen et al., 2001). To determine whether overexpression of BCL2 rescues the T cell developmental defects of human JAK3-deficient cells, we transduced in-vitro-derived, JAK3-deficient CD34^+^ cells with a lentivirus containing a BCL2–2A-GFP polycistron driven by the EF1a promoter. After transduction, CD34^+^ cells were plated onto OP9-DL4 monolayers and assayed for NK and T cell markers at T cell induction day 28. No CD3^−^CD16^+^CD56^+^ NK cells were found in GFP^−^ (JAK3^−^; BCL2 low) or GFP^+^ cells (JAK3^−^; BCL2^+^) (Figure 2C). These findings are consistent with reports demonstrating that the absence of NK cells in γc-deficient and Jak3-deficient mice is due to the lack of functional IL-15 signaling (Giri et al., 1994) and is independent of Bcl2-mediated anti-apoptosis. CD3^+^ cells were only detected in GFP^+^ (JAK3^−^; BCL2^+^) cells suggesting that BCL2 released the developmental block at the DN stage in JAK3-deficient cells. Interestingly, a second developmental arrest was evident at the DP stage; no further differentiation of CD8^+^CD4^+^ DP cells was observed in GFP^+^ cells (Figure 2C). These data are consistent with recent demonstrations that signaling by intrathymic IL-7 is necessary for CD8 lineage specification of DP thymocytes (Hare et al., 2000; Park et al., 2010).

In summary, the studies described above demonstrate that human SCID phenotypes can be recapitulated in vitro with patient-derived iPSCs. JAK3 deficiency results in proliferative defects in DN thymocytes. Forced expression of BCL2 enhances survival of DN cells, which further differentiate into DP thymocytes. Nevertheless, DP thymocytes fail to mature to SP T cells, and this defect may result from the absence of IL-7/JAK3 signaling.

### Correction of the JAK3 Deficiency in SCID Human Induced Pluripotent Stem Cells by CRISPR/Cas9-Enhanced Gene Targeting

**Figure 3.**
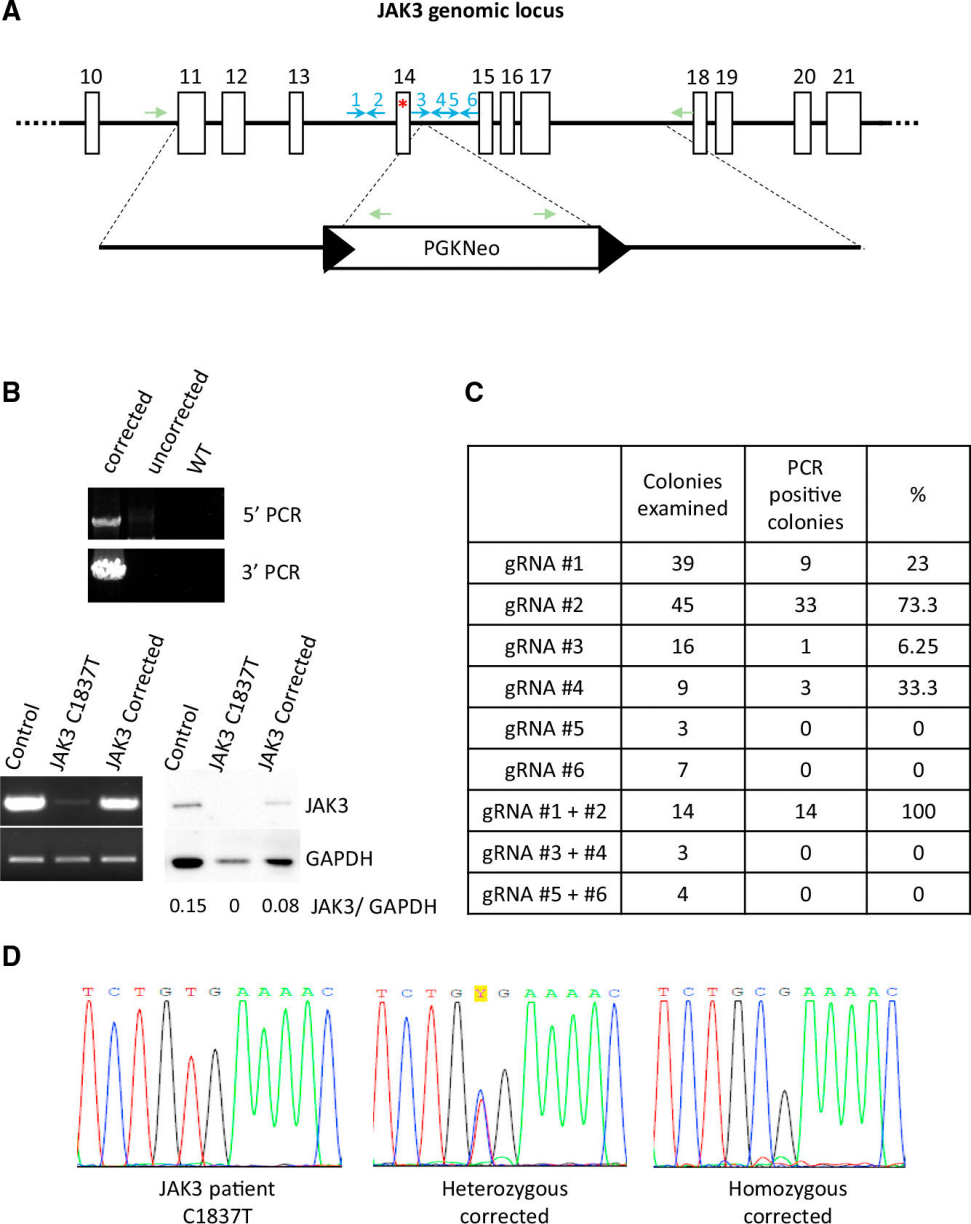
CRISPR/Cas9-Enhanced Correction of the JAK3 C1837T Mutation in Patient-Specific iPSCs (A) Strategy for genome modification using CRISPR/Cas9 to induce double-strand breaks in the JAK3 locus and a correction template for homology-directed repair. Top line, structure of the JAK3 gene. Open boxes, exons. Red asterisk, C1837T mutation. Blue arrows, guide RNAs. (B) (top) PCR analysis demonstrating homologous recombination; primers for 5′ and 3′ analysis are indicated by green arrows. (lower left) RT-PCR analysis demonstrating JAK3 mRNA expression in JAK3 WT (Control), JAK3-deficient (JAK3 C1837T), and corrected (JAK3 Corrected) T cells. (lower right) Western blot analysis demonstrating JAK3 protein expression in JAK3 WT (Control), JAK3-deficient (JAK3 C1837T), and corrected (JAK3 Corrected) T cells. JAK3/GAPDH ratios were quantified with ImageLab software (Bio-Rad). (C) Summary of targeting efficiencies of guide RNAs. (D) Sanger sequencing of the PCR amplicons from parental JAK3 iPSCs (left), heterozygous corrected (middle), and homozygous corrected iPSCs (right). The heterozygous clone was corrected with wild-type Cas9 + gRNA#2, and the homozygous clone was corrected with D10A Cas9 nickase + gRNA#1 and #2.

To determine whether normal T cell development can be restored in JAK3-deficient SCID patient cells, we corrected the JAK3 mutation in iPSCs by CRISPR/Cas9-enhanced gene targeting. Six guide RNAs within introns upstream and downstream of exon 14 were designed to target wild-type Cas9 or the D10A Cas9 nickase near the C1837T mutation, and a correction template was used for homology-directed repair (HDR) (Figure 3A). IPSCs were nucleofected with two plasmids expressing the D10A Cas9 nickase and paired guide RNAs or a single plasmid expressing wild-type Cas9 and a single guide RNA. Cells were grown in medium containing G418 for 2 weeks post nucleofection. Individual colonies were picked, expanded, and genotyped by PCR (Figure 3B, top). The efficiency of CRISPR/Cas9-mediated JAK3 gene correction is shown in Figure 3C. Three clones from wild-type Cas9 + gRNA #1, three clones from wild-type Cas9 + gRNA #2, and six clones from D10A Cas9 nickase + paired gRNAs #1 and #2 were further verified by Sanger sequencing. In 12 sequenced clones, two homozygous corrected clones (one clone from D10A Cas9 nickase + paired gRNA #1 and #2 and one clone from wild-type Cas9 + gRNA #1) and ten heterozygous corrected clones were identified (Figure 3D). Restoration of JAK3 gene expression was demonstrated by RT-PCR (JAK3 mRNA) (Figure 3B, lower-left panel) and western blot (JAK3 protein) (Figure 3B, lower right).

### Specificity of CRISPR/Cas9-Directed JAK3 Correction

The potential for off-target, CRISPR/Cas9-directed genome modifications raises some concerns about the use of this approach for therapy in humans. In cancer cell lines, relatively high levels of off-target mutagenesis by Cas9-gRNAs have been described (Fu et al., 2013). However, several groups have recently demonstrated by whole-genome sequencing (WGS) that off-target modifications are rare in human iPSCs and human embryonic stem cells (Smith et al., 2014; Veres et al., 2014). To determine the specificity of CRISPR/Cas9-directed JAK3 correction in human SCID iPSCs, we performed WGS before and after gene correction. The genomes of two heterozygous and one homozygous corrected clones were sequenced. The two heterozygous clones were corrected with gRNA #2 + wild-type Cas9, and the homozygous clone was corrected with gRNA #1 + gRNA #2 + D10A nickase Cas9.

The 20-base CRISPR guide sequences were mapped to the human reference genome, allowing up to three mismatches in order to identify potential off-target sites (Table 1; Figure S1). These sites were then analyzed for variations in the iPSC samples following CRISPR/Cas9-directed gene correction. Whole-genome sequencing of the one homozygous and two heterozygous corrected iPSC lines demonstrated that no mutations (SNVs nor indels) were introduced into the 1,450 potential off-target sites (Table 2). These results demonstrate the specificity of CRISPR/Cas9-directed gene correction.

### Restoration of T Cell Development after CRISPR/ Cas9-Directed JAK3 Correction

**Figure 4.**
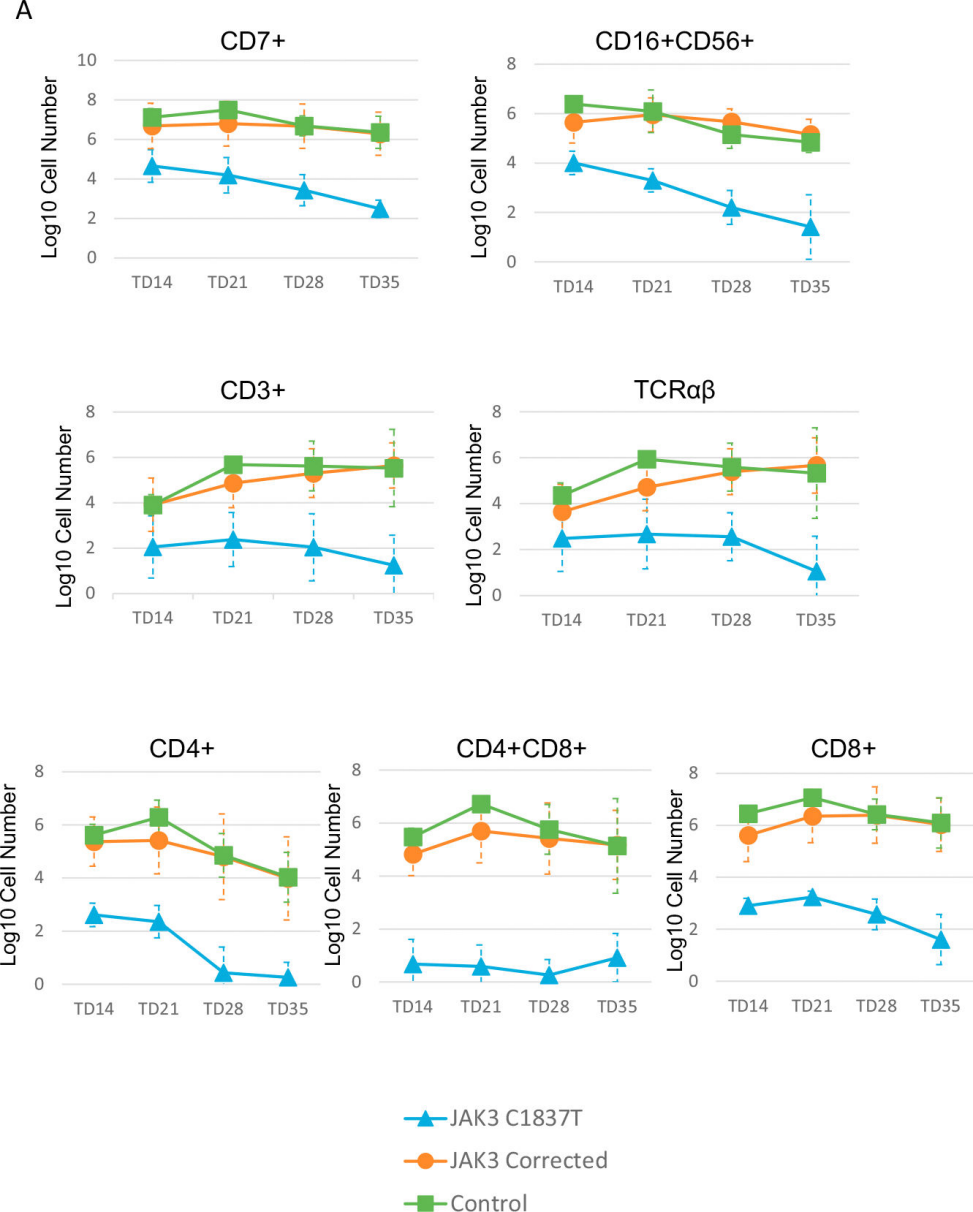
In Vitro Differentiation of JAK3-Corrected Patient iPSCs Produces NK Cell and T Cells with Phenotypic and Functional Characteristics of Mature T Cells Expression of NK cell and T cell developmental markers in JAK3 WT (Control, green, n = 3), JAK3-deficient (JAK3 C1837T, blue, n = 5), and JAK3-corrected (JAK3 Corrected, orange, n = 6) T cells. Cells were stained with the indicated antibodies and analyzed by flow cytometry at T cell induction days 14, 21, 28, and 35 (TD 14, 21, 28 and 35). Data are shown as the mean ± SD.

To determine whether T cell development is restored after JAK3 gene correction, NK and T cell generation were verified, and T cell lineage commitment and maturation were analyzed. We and other groups have previously demonstrated in the OP9-DL4 in vitro system that T cell differentiation sequentially passes through intermediates observed in vivo: CD34^+^CD7^+^ T/NK committed stage; CD7^+^CD4^+^CD8^−^ immature, SP stage; CD4^+^CD8^+^ DP stage; and finally, CD3^+^CD8^+^ TCRαβ mature stage. Mature T cells are polyclonal, proliferate, and secrete cytokines in response to mitogens (Chang et al., 2014; Timmermans et al., 2009). Therefore, control, JAK3-deficient, and JAK3-corrected human iPSCs were differentiated into hematopoietic progenitors on OP9 monolayers, and CD34^+^ cells were positively selected with anti-CD34 magnetic beads. These cells were plated onto OP9-DL4 monolayers, and non-adherent cells were analyzed for lymphocyte markers at T cell induction day (TD) 14, 21, 28, and 35 (Figure 4). In normal controls (green line), 1.2 × 10^7^ CD7^+^ cells (84% of cells counted in the lymphoid gate) were generated at T cell induction day 14 from 1–2 × 10^6^ CD34^+^ cells. At this stage, about 20% of CD7^+^ cells were CD7^+^CD16^+^CD56^+^ NK cells (2.4 × 10^6^). T cell markers CD4, CD8, CD3, and TCR αβ were sequentially detected upon T cell maturation. As we reported previously, at T cell induction day 35 the NK population decreased (6.9 × 10^4^), and more than 50% of residual cells were CD8 SP cells (1.2 × 10^6^). In JAK3-deficient cells (blue line), only 4.5 × 10^4^ CD7^+^ cells (38.9% of cells counted in lymphoid gate) were generated at T cell induction day 14 from 1–2 × 10^6^ CD34^+^ cells. The number of total CD7^+^ cells decreased during extended culture and T cell markers CD3, CD4, CD8, and TCR αβ were not significantly expressed.

Similar to control cells, 1–2 × 10^6^ CD34^+^ JAK3-corrected cells differentiated into 4.7 × 10^6^ CD7^+^ cells (91% of cells counted in lymphoid gate) at T cell induction day 14 including 4.3 × 10^5^ CD7^+^CD16^+^CD56^+^ NK cells. After further differentiation to TD21, TD28, and TD35, T cell maturation markers CD3, CD4, CD8, and TCR αβ were abundantly observed (Figure 4). Our data indicate that JAK3-corrected iPSCs generate NK and T cells with similar efficiency.

**Figure 5.**
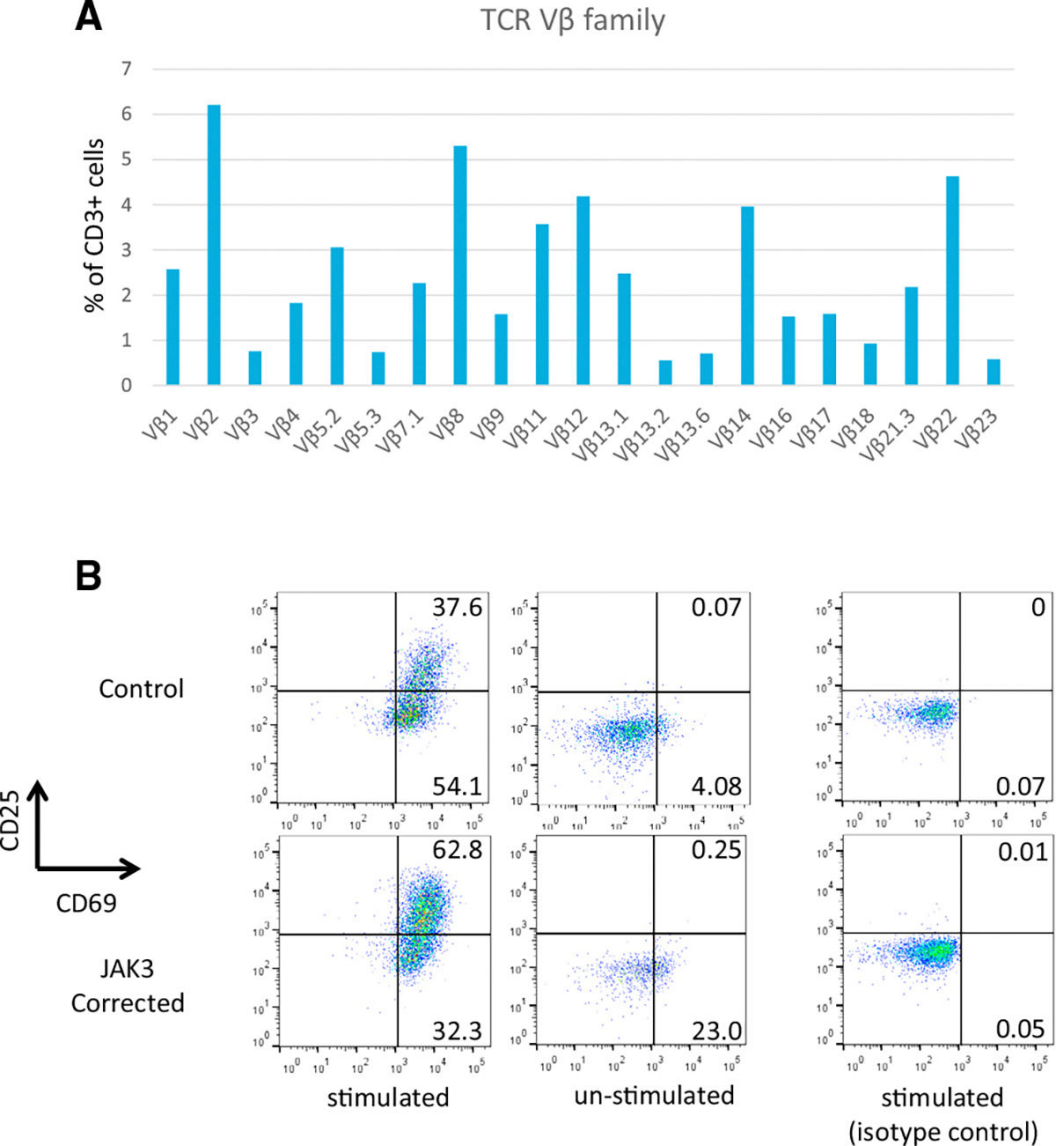
In Vitro Differentiation of JAK3-Corrected Patient iPSCs Produces T Cells with Phenotypic and Functional Characteristics of Mature T Cells (A) T cell receptor (TCR) Vβ analysis of JAK3-corrected T cells. A highly diverse repertoire of TCR Vβ is represented in T cells derived from corrected SCID patient iPSCs. (B) Flow cytometry demonstrating T cell activation in JAK3-corrected T cells. T cells derived from JAK3 WT (Control) and JAK3-corrected iPSCs were stimulated with anti-CD3/28 beads for 3 days before analysis of activation markers CD25 and CD69. Matched isotype antibodies were used as negative controls (isotype). These data were gated on CD3^+^ populations.

To determine whether TCR rearrangement is reestablished in JAK3-corrected T cells, TCR Vβ typing was performed by flow cytometry and summarized in Figure 5A. JAK3-corrected T cells expressed all 21 of the Vβ segments that we tested therefore, a broad TCR repertoire was restored. Finally, we examined the integrity of the TCR signaling pathway, a surrogate of T cell function, in JAK3-corrected T cells by measuring cell surface activation markers following anti-CD3/CD28 stimulation. On day 3 post-stimulation, the percentage of CD3^+^CD25^+^CD69^+^ T cells increased from 0.68% to 59.7% in JAK3-corrected T cells, similar to the increase observed in control cells (0.01%–37.6%) (Figure 5B). These data and results described above demonstrate that correction of the JAK3 C1837T (p.R613X) mutation by CRISPR/Cas9-enhanced gene targeting in an in vitro iPSC model system restores normal T cell development with the capacity to produce functional, mature T cell populations with a broad TCR repertoire.

## DISCUSSION

Much has been learned about lymphopoiesis in the past 2 decades from naturally occurring immunodeficient mice and from knockout and knockin mouse models. These data have provided fundamental knowledge about the mechanisms involved in lymphocyte development and activity. In humans, the phenotype of lymphocytes in the peripheral blood of SCID patients has been well described, but studies on critical steps of lymphoid commitment and thymocyte development have been difficult to perform. Access to bone marrow and thymocyte samples from untreated patients with SCID is challenging since these conditions are rare and infants typically present with life-threatening infections requiring urgent HSC transplantation to survive. The strategy that wedescribefor studyinghuman SCIDbypassesthese restrictions; large numbers of hematopoietic progenitors can be produced from patient-specific iPSCs in vitro, and the mechanisms responsible for immunodeficiency can be precisely determined. In this study, we demonstrate that T cell development in human JAK3-deficient SCID is completely blocked before or at the CD4^−^CD8^−^ (DN2) stage. Interestingly, forced expression of BCL2 enhances survival of DN cells, which further differentiate into DP thymocytes. However, DP thymocytes fail to mature to SP T cells, and this defect may result from the absence of IL-7/JAK3 signaling.

We also demonstrate that correction of the human JAK3 mutation by CRISPR/Cas9-enhanced gene targeting restores the differentiation potential of early T cell progenitors. Corrected progenitors are capable of producing NK cells and mature T cell populations expressing a broad TCR repertoire. Whole-genome sequencing of one homozygous and two heterozygous corrected iPSC lines demonstrates that no mutations (SNVs nor indels) are introduced into 1,450 potential off-target sites, suggesting a strong specificity for CRISPR/Cas9-directed gene correction. In summary, these studies describe an approach for the study of human lymphopoiesis and provide a foundation for gene correction therapy in humans with immunodeficiencies and other monogenic disorders. For gene therapy, we envision transplantation of the CD34^+^ cells that are generated in the first phase of in vitro culture. These cells include early multipotent hematopoietic progenitors that generate all myeloid and erythroid cells in colony forming assays in addition to the lymphoid cells that we describe here. Our results suggest that there are no intrinsic defects in lineage specification of early hematopoietic progenitors produced in vitro. However, we have not been able to generate all of these lineages after transplantation into immunodeficient (NSG) mice. These results suggest that human hematopoietic progenitors produced in vitro do not home to mouse bone marrow niches that support self-renewal. However, these early progenitors may be incorporated into human bone marrow niches to which endogenous progenitors naturally home. After safety studies are completed in NSG mice, phase 1 clinical trials will be required to determine whether these early progenitors are capable of engraftment and sustained reconstitution of multiline-age hematopoiesis in human patients.

## EXPERIMENTAL PROCEDURES

### Patient Information

The patient was enrolled in an institutional review board-approved study, and parents signed consents, in accordance with the Declaration of Helsinki. The family history was negative for immune deficiencies. For the first 8 months of age, he had poor weight gain, diarrhea, and recurrent bronchiolitis requiring frequent hospitalization. He was admitted to the hospital at 8 months of age with severe respiratory distress and oral thrush. Bronchoscopy with bronchial alveolar lavage demonstrated bacterial (pseudomonas, H flu, S. pneumonia) and viral organisms (respiratory syncytial virus). Immunologic evaluations demonstrated severe hypogammaglobulinemia, with an immunoglobulin E (IgE) <3 μl/ml, IgA <4 mg/dl, IgG = 29 mg/dl, IgM = 26 mg/dl. Immune phenotyping of peripheral blood demonstrated complete absence of CD3^+^ T cells and NK cells, though B cells were present (absolute B cell count = 875 μl). Mitogen studies demonstrated a complete lack of response to concanavalin A, pokeweed mitogen and phytohemagglutinin A. The diagnosis of SCID was confirmed by genetic testing, which indicated a homozygous C > T nucleotide substitution in exon 14 of the JAK3 gene, resulting in the replacement of an arginine codon (CGA) with a stop codon (TGA) at amino acid position 613. This is the first report linking this JAK3 variant (rs149316157) to a clinical case of SCID. The patient underwent a reduced intensity conditioning matched unrelated bone marrow transplant and is doing well now two years off therapy with complete immune reconstitution.

### Human iPSC Reprogramming and Characterization

For iPSC induction, 5 × 10^4^ primary keratinocytes were seeded into one well of a 6-well plate. On the following day, keratinocytes were transduced with 1 ml of virus supernatant and 1 ml of human keratinocyte medium containing polybrene at a final concentration of 4 μg/ml. The keratinocytes were spinfected at 800 × g for 45 min (day 1). The transduction procedure was repeated again the next day. On day 3, cells were changed to fresh human keratinocyte medium and cultured for 2 more days. Onday 5, the keratinocytes were trypsinized and transferred to a 10-cm dish pre-seeded with mitomycin C-treated murine embryonic fibroblasts (MEFs) and cultured in human keratinocyte medium. On day 7, cells were changed to human embryonic stem (ES) medium and continuously cultured on the same dish for 3–4 weeks. ES medium was changed daily. Potential iPSC colonies were visible after 2–3 weeks. These colonies were individually picked and expanded on MEFs for analysis. To remove the integrated lentiviral and polycistronic sequences, iPSCs were infected with a Cre-expressing adenovirus (rAd-Cre-IE). Individual colonies were picked and Cre-mediated removal of floxed sequences was verified by PCR using the primers gctaattcactcccaaagaagacaag and cttcagcaagccgagtcctg (Figure S2).

### Generation of CD34^+^ Cells and T Cells with OP9 Co-culture

The procedure was described previously (Chang et al., 2014) with the following modifications. Cultures of human induced pluripotent stem cells (hiPSCs) in one well of 6-well plate were treated as described by Ohnuki and Yamanaka (2009) with collagenase-trypsin-knockout serum replacement (CTK-KSR) solution to make small cell clumps. Cell clumps were then transferred to a 10-cm plate that was pre-seeded with 2-day old OP9 cells in a-MEM-based medium containing 10% FBS, 1 × penicillin/streptomycin and 100 mM mono-thioglycerol. The medium was changed every other day, and cells were cultured for 18 days without any splitting. After 18 days of co-culture, cells were harvested by treating with dissociation solution (0.15% collagenase IV and 0.015% hyaluronidase in α-MEM medium) for about 30 min and followed by 0.25% trypsin for another 30 min. CD34^+^ cells were then purified on anti-CD34^+^ magnetic beads (MicroBead Kit; Miltenyi Biotec). For T cell differentiation, these CD34^+^ cells were plated onto OP9-DL4 cells and cultured with α-MEM medium containing 20% FBS, 5 ng/ml hFlt3-L, 5 ng/ml hIL-7, and 10 ng/ml hSCF. The medium was changed every other day, and cells were transferred to new OP9-DL4 plates every 4 days. After completion of our studies, Menon et al. (2015) described the correction of SCID-X1 patient-derived iPSCs by TALENs and the generation of mature NK cells and T cell precursors.

### T Cell Stimulation

In vitro derived T cells from hiPSCs were stimulated by incubation with CD3/28 beads (Invitrogen) according to the manufacturer’s protocol for 3 days prior to analysis by flow cytometry, as previously described (Chang et al., 2014).

### Flow Cytometry

Cells were harvested and washed before analysis with an LSRFortessa cell analyzer (BD Bioscience). For cell surface staining, propidium iodide (PI, Sigma-Aldrich) was used to exclude dead cells. For the apoptosis assay, harvested cells were first stained with cell surface antibodies for 30 min. After washing once with 1 × PBS, the cells were resuspended in 100 μl of Annexin Binding Buffer (Invitrogen) containing Annexin V-647 (Invitrogen) and PI and incubated for 15 min before adding 400 ml of Annexin Binding Buffer with PI. Antibodies were obtained from BD Biosciences unless otherwise indicated: CD3 (Percp-Cy5–5, clone UCHT1), CD4 (PE-Cy7, clone SK3), CD7 (APC, BV510, clone M-T701), CD8 (APC-Cy7, clone SK1), CD16 (PE, clone B73.1), CD25 (FITC, clone 2A3), CD34 (PE-Cy7, clone WM59), CD43 (PE, clone 1G10), CD56-PE (clone MY31), CD69 (FITC, clone L78), TCR-αβ (FITC, PE, clone T10B9.1A-31), Beta Mark TCR Repertoire Kit (Beckman Coulter).

### Vector Construction

The polycistronic OSKM vector was previously described (Chang et al., 2009). The Lenti-hDL4-mCherry plasmid was constructed by cloning a PCR-amplified human DL4 cDNA (Open Biosystems), an IRES fragment (Open Biosystems) and mCherry cDNA into a lentiviral vector (pDL171) which contains the EF1a promoter. PCRs were performed using PrimeStar polymerase (Takara). To construct CRISPR plasmids, we cloned designed gRNA oligos into pX330 and pX335 plasmids following the Zhang lab protocol (Addgene). The list of primers for construction of gRNA sequences is as follows: (1) gRNA-F1: caccGTG AGA TAC AGA TAC AGA CA, (2) gRNA-R1: aaacTGT CTG TAT CTG TAT CTC AC, (3) gRNA-F2: caccgAAT GAT TTG CCT GGA ATG CC, (4) gRNA-R2: aaacGGC ATT CCA GGC AAA TCA TTc, (5) gRNA-F3: caccg CAG CCT AGG CAA AGG CCT GC, (6) gRNA-R3: aaacGCA GGC CTT TGC CTA GGC TGc, (7) gRNA-F4: caccgTGC CAA CAG AAC TGC CTG AT, (8) gRNA-R4: aaacATC AGG CAG TTC TGT TGG Cac, (9) gRNA-F5: caccGAC CAG GGT GCA AGT GTG GA, (10) gRNA-R5: aaacTCC ACA CTT GCA CCC TGG TC, (11) gRNA-F6: caccGCT CCT CAG CCT GGC ATT CA, and (12) gRNA-R6: aaacTGA ATG CCA GGC TGA GGA GC. To construct the JAK3 repair plasmid, wild-type human genomic DNA was PCR amplified using JAK3 primer sets (5′ arm, forward: gtcgacgtcgacgctcagtgaagctgaagtattcctt ctgcttcacagggcgaccactac and 5′ arm, reverse: atttaaatcctcccctcgaacccttac caaactcctatgcatactacag; 3′ arm, forward: ttaattaattaattagcattttaggttcaggttgt gagaacactagaagagaacaagtca and 3′ arm, reverse: gtatacgtatacgcatacctg gagaggggacaaggtcttgagatgcgagggt).

After digesting with enzymes (5′ arm, SalI and SwaI; 3′ arm, PacI and BstZ17I), the PCR products were cloned into a plasmid containing a LoxP-PGK-Neo-LoxP fragment. All of the oligos used in this study were synthesized by Integrated DNA Technologies (IDT). To construct the BCL2 lentiviral plasmid, a primer set (forward: agccaccttaattaagccaccatggcgcacgctggga gaacggggtacgata and reverse: taacagagagaagttcgtggctccggatcccttgtggcc cagataggcacccagggtgat) was used to amplify the human BCL2 cDNA (Open Biosystems) fragment. The amplified product was linked to GFP through a 2A sequence by PCR and cloned into the pDL171 vector.

### Cell Culture

IPSCs were cultured on mitomycin-C-treated MEFs derived from E14.5 CF-1 embryos in ES cell medium consisting of DMEM F-12 supplemented with 1 × non-essential amino acids, 1 × penicillin-streptomycin, 1 × L-glutamine (all from Mediatech), 20% Knockout Serum Replacement (Invitrogen), 2-ME (Sigma), and 5–10 ng/ml bFGF (Invitrogen). Human primary keratinocytes were cultured in DermaLife K Medium Complete Kit (LifeLine Cell Technology). OP9 cells were purchased from ATCC and grown in α-MEM medium with 20% FBS and penicillin-streptomycin. OP9-DL4 cells were established by transducing OP9 cells with a lentivirus containing hDL4 and mCherry.

### Virus Production

For preparation of lentivirus, 10 mg of the lentiviral vector, 2.5 mg of the envelope plasmid (pMDG), and 7.5 mg of the packaging plasmid (pCMBVdR8.9.1) were co-transfected into 5 × 10^6^ 293T cells by Fugene 6 (Roche or Promega). Virus-containing supernatant was collected 2 days after transfection and passed through a 0.45-mm filter.

### Gene Targeting

IPSCs were treated with 0.25% trypsin for 5 min to generate single-cell suspensions. After washing twice with 1 × PBS, 1–2 million cells were mixed with 5 μg of JAK3 repair plasmid and 5 μg of either pX330-JAK3 or pX335-JAK3 plasmid for Nucleofection (Human Stem Cell Nucleofector Kit, program A-023, Lonza) and subsequent plating onto MEFs. Two to 4 days later, hES medium containing 30 μg/ml of G418 was added to the plates to select for drug-resistant colonies. The colonies were picked 3–4 weeks post selection and expanded for genomic DNA extraction. For genotyping, a 5′ primer set (tgctaaagcgcatgctccagact and gtcttcatctcagggtcggct) and a 3′ primer set (cctctctgtgcattatggcag and gccttctatcgccttcttg) were used. To remove the Neo selection marker, iPSCs were infected with a Cre-expressing adenovirus (rAd-Cre-IE) and later analyzed by PCR for marker loss using the following primers: Jak3-Creout-F ttgggagtgggctctgtagtatgc and Jak3-Creout-R ttcttcctgcccagcctcgtcatt (Figure S2).

### RT-PCR

Total RNA was isolated from in-vitro-derived cells with Trizol reagent (Invitrogen). cDNA was synthesized from 0.5–2 μg of total RNA using the Superscript First-strand Synthesis System (Invitrogen) according to the manufacturer’s instructions. SYBR Green PCR Master Mix (Life Technologies) was used for qPCR according to the manufacturer’s instructions. All values were normalized relative to GAPDH expression. Primer sets used for qPCR are GAPDH (F: actcctccacctttgacgct, R: tcccctcttcaagggtctacatg); PU.1 (F: gtgcaaaatggaag ggtttc, R: ggagctccgtgaagttgttc); GATA3 (F: tgtttcctttcactggccaca, R: aacggc aactggtgaacggta); BCL11B (F: ggcgatgccagaatagatgccg, R: ccaggccacttggc tcctctatctccaga); RAG1 (F: ccttactgttgagactgcaatatcc, R: ctgaagtcccagtatat acttcacac); RAG2 (F: cccagaagcagtaataatcatcgag, R: atgtgggatgtagtagatc ttgc); pTa (F: gggtcttacctcagcagttac, R: cctcacacagtgtgacgcag); BCL2 (F: gactgagtacctgaaccggc, R: gggccaaactgagcagagtc); BAX (F: aagaccagggtg gttgggac, R: gtaagaaaaatgcccacgtc); and JAK3 (F: agtcagacgtctggagcttc, R: gtgagcagtgaaggcatgagtc).

### Whole-Genome Sequencing and Analysis

DNA from iPSCs was sheared using a Covaris S2 Focused-ultrasonicator: 130-μl samples in microTUBEs were subjected to two 60-s cycles of 10% duty cycle, intensity of 4, and 200 cycles per burst in frequency sweeping mode. DNA Chip (DNA 1000 Kit; Agilent Technologies) analysis using an Agilent 2100 Bioanalyzer indicated an average fragment size of 400 bp. Library preparation was performed using an NEBNext Ultra DNA Library Prep Kit for Illumina (NEB #E7370), and the final library concentration was determined by qPCR using a KAPA Illumina Library Quantification Kit (KK4835; KAPA Biosystems) and an Applied Biosystems ViiA 7 Real-Time PCR System (Life Technologies).

Sequencing clusters were produced on the flow cell using an Illumina TruSeq PE Cluster Kit v.3-cBot-HS (PE-401–3001) and an Illumina cBot. WGS was performed using an Illumina TruSeq SBS Kit v.3-HS-200 cycles (FC-401–3001) and an Illumina HiSeq 2500 upgrade to generate 2 × 100 single-index paired-end reads for bioinformatic analysis.

Potential off-target sites were identified by aligning the CRISPR/Cas9 guide sequences to the hg19 reference genome using EMBOSS fuzznuc software (v.6.6.0.0) (Rice et al., 2000) and allowing for a maximum of three mismatches; 1,193 sites were identified for the first guide sequence (GTGAGATACAGATA CAGACA) and 257 sites for the second guide sequence (AATGATTTG CCTGGAATGCC).

All of the reads from the WGS for each sample were mapped to the hg19 reference genome using the BWA (v.0.7.5a) mem algorithm (Li and Durbin, 2010) and duplicate reads were removed using Picard tools (v.1.100) (http://broadinstitute.github.io/picard/). Local realignment and base quality re-calibration were performed using GATK (v.2.7–2) (McKenna et al., 2010). Both SNVs and indels were called using GATK HaplotypeCaller. Additionally, SNVs and indels were separately re-calibrated as described in GATK Best Practices, and quality filters were applied. Genome STRiP v.2.0 (Handsaker et al., 2011, 2015) was used to call the large deletions against a background population of 100 samples from the 1000 Genomes Project. Inversions, duplications, and translocations were called using Delly v.0.6.3 (Rausch et al., 2012), and insertions were called using Pindel v.0.2.5a8 (Ye et al., 2009).

Variants from the reference genome that were common to all four iPSC samples were excluded from CRISPR/Cas9 off-target analysis. The non-excluded variants were screened using BEDTools (v.2.17.0) (Quinlan and Hall, 2010) to determine whether they fell within the potential off-target sites ±100 flanking base pairs. The analysis demonstrated that none of these variants reside in the off-target sites and suggests that these variants were randomly accumulated.

All of the functional variants (excluded and non-excluded) with a low allele frequency (<1% in dbSNP 138, 1000 Genomes, and NHLBI-ESP 6500), conserved in phastCons 46-way elements (Siepel et al., 2005) and with a high CADD score (CADD R10) (Kircher et al., 2014) were then annotated using the ANNOVAR software package (Wang et al., 2010) and screened for known associations with diseases in ClinVar (v.20140902) (Landrum et al., 2014), GWAS Catalog (Welter et al., 2014), and COSMIC (v.70) (Forbes et al., 2008); additionally, we manually screened the variants with a high CADD score (CADD >20) in HGMD (Stenson et al., 2003). None of these variants, including the JAK3 C1837T variant, were found to be associated with disease in the databases queried. Unlike the other variants, however, the JAK3 C1837T (p.R613X) variant (rs149316157) is predicted to be significantly deleterious, with a GERP score (Davydov et al., 2010) of 3.85 and a CADD score of 36; we report here the association of this JAK3 variant with a clinical case of SCID.

## Supplementary Material

Data Supplement

SUPPLEMENTAL INFORMATION

Supplemental Information includes two figures and can be found with this article online at http://dx.doi.org/10.1016/j.celrep.2015.08.013.

## Figures and Tables

**Table 1.** Identification of Potential Off-Target Sites

	gRNA#l	gRNA#2
Sequence	GTGAGATACAGATACAGACA	AATGATTTGCCTGGAATGCC
Target sites (0 mismatch)	1	1
1 base mismatch, potential off-target sites	3	0
2 base mismatch, potential off-target sites	80	13
3 base mismatch, potential off-target sites	1,109	243
Total	1,193	257

Potential off-target sites were identified by aligning the CRISPR/Cas9 guide sequences to the hg19 reference genome using EMBOSS fuzznuc software (v.6.6.0.0) (Rice et al., 2000) and allowing for a maximum of three mismatches.

**Table 2.** Variant Analysis of Potential Off-Target Sites

	Clone 1	Clone 2	Clone 3
Cas9	wild-type	wild-type	D10A nickase
gRNA	gRNA#2	gRNA#2	gRNA#l+gRNA#2
JAK3 C1837T genotype	C/T	C/T	C/C
Discordant variants in off-targets and flanking 100 bases	0	0	0

Whole-genome sequencing (WGS) was performed on three corrected iPSC clones and the uncorrected control (see Whole-Genome Sequencing and Analysis in Experimental Procedures). Variants (from the reference genome) that were common to all four iPSC samples were excluded from further analysis. The remainder were screened to determine whether these variants were located in potential off-target sites. No variants were observed in potential off-target sites. BEDTools (v.2.17.0) (Quinlan and Hall, 2010) was used to search for non-excluded variants in potential off-target sites.

## Highlights

- iPSC modeling of SCID
- CRISPR/Cas-enhanced gene correction
- Broad T cell receptor repertoire
- No off-target modifications

## References

[R1] Aiuti A, Cattaneo F, Galimberti S, Benninghoff U, Cassani B, Callegaro L, Scaramuzza S, Andolfi G, Mirolo M, Brigida I, (2009). Gene therapy for immunodeficiency due to adenosine deaminase deficiency. N. Engl. J. Med. 360, 447–458. 19179314 10.1056/NEJMoa0805817

[R2] Biffi A, Montini E, Lorioli L, Cesani M, Fumagalli F, Plati T, Baldoli C, Martino S, Calabria A, Canale S, (2013). Lentiviral hematopoietic stem cell gene therapy benefits metachromatic leukodystrophy. Science 341, 1233158. 10.1126/science.1233158 23845948

[R3] Chang CW, Lai YS, Pawlik KM, Liu K, Sun CW, Li C, Schoeb TR, and Townes TM (2009). Polycistronic lentiviral vector for ‘‘hit and run’’ reprogramming of adult skin fibroblasts to induced pluripotent stem cells. Stem Cells 27, 1042–1049. 19415770 10.1002/stem.39

[R4] Chang CW, Lai YS, Lamb LS Jr., and Townes TM (2014). Broad T-cell receptor repertoire in T-lymphocytes derived from human induced pluripotent stem cells. PLoS ONE 9, e97335. 10.1371/journal.pone.0097335 PMC4020825 24828440

[R5] Cong L, Ran FA, Cox D, Lin S, Barretto R, Habib N, Hsu PD, Wu X, Jiang W, Marraffini LA, and Zhang F (2013). Multiplex genome engineering using CRISPR/Cas systems. Science 339, 819–823. 23287718 10.1126/science.1231143 PMC3795411

[R6] Davydov EV, Goode DL, Sirota M, Cooper GM, Sidow A, and Batzoglou S (2010). Identifying a high fraction of the human genome to be under selective constraint using GERP++. PLoS Comput. Biol. 6, e1001025. 10.1371/journal.pcbi.1001025 PMC2996323 21152010

[R7] de Pooter R, and Zúñiga-Pflücker JC (2007). T-cell potential and development in vitro: the OP9-DL1 approach. Curr. Opin. Immunol. 19, 163–168. 17303399 10.1016/j.coi.2007.02.011

[R8] Dervovic DD, Ciofani M, Kianizad K, and Zúñiga-Pflücker JC (2012). Comparative and functional evaluation of in vitro generated to ex vivo CD8 T cells. J. Immunol. 189, 3411–3420. 22925927 10.4049/jimmunol.1200979

[R9] Eynon EE, Livák F, Kuida K, Schatz DG, and Flavell RA (1999). Distinct effects of Jak3 signaling on alphabeta and gammadelta thymocyte development. J. Immunol. 162, 1448–1459. 9973401

[R10] Ferrua F, Brigida I, and Aiuti A (2010). Update on gene therapy for adenosine deaminase-deficient severe combined immunodeficiency. Curr. Opin. Allergy Clin. Immunol. 10, 551–556. 20966749 10.1097/ACI.0b013e32833fea85

[R11] Forbes SA, Bhamra G, Bamford S, Dawson E, Kok C, Clements J, Menzies A, Teague JW, Futreal PA, and Stratton MR (2008). The catalogue of somatic mutations in cancer (COSMIC). Curr. Protoc. Hum. Genet. Chapter 10, Unit 10.11. 10.1002/0471142905.hg1011s57. PMC2705836 18428421

[R12] Fu Y, Foden JA, Khayter C, Maeder ML, Reyon D, Joung JK, and Sander JD (2013). High-frequency off-target mutagenesis induced by CRISPR-Cas nucleases in human cells. Nat. Biotechnol. 31, 822–826. 23792628 10.1038/nbt.2623 PMC3773023

[R13] Giri JG, Ahdieh M, Eisenman J, Shanebeck K, Grabstein K, Kumaki S, Namen A, Park LS, Cosman D, and Anderson D (1994). Utilization of the beta and gamma chains of the IL-2 receptor by the novel cytokine IL-15. EMBO J. 13, 2822–2830. 8026467 10.1002/j.1460-2075.1994.tb06576.x PMC395163

[R14] Hacein-Bey-Abina S, Von Kalle C, Schmidt M, McCormack MP, Wulffraat N, Leboulch P, Lim A, Osborne CS, Pawliuk R, Morillon E, (2003). LMO2-associated clonal T cell proliferation in two patients after gene therapy for SCID-X1. Science 302, 415–419. 14564000 10.1126/science.1088547

[R15] Hacein-Bey-Abina S, Garrigue A, Wang GP, Soulier J, Lim A, Morillon E, Clappier E, Caccavelli L, Delabesse E, Beldjord K, (2008). Insertional oncogenesis in 4 patients after retrovirus-mediated gene therapy of SCID-X1. J. Clin. Invest. 118, 3132–3142. 18688285 10.1172/JCI35700 PMC2496963

[R16] Hacein-Bey-Abina S, Pai SY, Gaspar HB, Armant M, Berry CC, Blanche S, Bleesing J, Blondeau J, de Boer H, Buckland KF, (2014). A modified g-retrovirus vector for X-linked severe combined immunedeficiency. N. Engl. J. Med. 371, 1407–1417. 25295500 10.1056/NEJMoa1404588 PMC4274995

[R17] Handsaker RE, Korn JM, Nemesh J, and McCarroll SA (2011). Discovery and genotyping of genome structural polymorphism by sequencing on a population scale. Nat. Genet. 43, 269–276. 21317889 10.1038/ng.768 PMC5094049

[R18] Handsaker RE, Van Doren V, Berman JR, Genovese G, Kashin S, Boettger LM, and McCarroll SA (2015). Large multiallelic copy number variations in humans. Nat. Genet. 47, 296–303. 25621458 10.1038/ng.3200 PMC4405206

[R19] Hanna J, Wernig M, Markoulaki S, Sun CW, Meissner A, Cassady JP, Beard C, Brambrink T, Wu LC, Townes TM, and Jaenisch R (2007). Treatment of sickle cell anemia mouse model with iPS cells generated from autologous skin. Science 318, 1920–1923. 18063756 10.1126/science.1152092

[R20] Hare KJ, Jenkinson EJ, and Anderson G (2000). An essential role for the IL-7 receptor during intrathymic expansion of the positively selected neonatal T cell repertoire. J. Immunol. 165, 2410–2414. 10946265 10.4049/jimmunol.165.5.2410

[R21] Kang J, Coles M, and Raulet DH (1999). Defective development of gamma/delta T cells in interleukin 7 receptor-deficient mice is due to impaired expression of T cell receptor gamma genes. J. Exp. Med. 190, 973–982. 10510087 10.1084/jem.190.7.973 PMC2195640

[R22] Kircher M, Witten DM, Jain P, O’Roak BJ, Cooper GM, and Shendure J (2014). A general framework for estimating the relative pathogenicity of human genetic variants. Nat. Genet. 46, 310–315. 24487276 10.1038/ng.2892 PMC3992975

[R23] Kondo M, Akashi K, Domen J, Sugamura K, and Weissman IL (1997). Bcl-2 rescues T lymphopoiesis, but not B or NK cell development, in common gamma chain-deficient mice. Immunity 7, 155–162. 9252128 10.1016/s1074-7613(00)80518-x

[R24] Landrum MJ, Lee JM, Riley GR, Jang W, Rubinstein WS, Church DM, and Maglott DR (2014). ClinVar: public archive of relationships among sequence variation and human phenotype. Nucleic Acids Res. 42, D980–D985. 24234437 10.1093/nar/gkt1113 PMC3965032

[R25] Li H, and Durbin R (2010). Fast and accurate long-read alignment with Burrows-Wheeler transform. Bioinformatics 26, 589–595. 20080505 10.1093/bioinformatics/btp698 PMC2828108

[R26] Li WQ, Jiang Q, Khaled AR, Keller JR, and Durum SK (2004). Interleukin-7 inactivates the pro-apoptotic protein Bad promoting T cell survival. J. Biol. Chem. 279, 29160–29166. 15123689 10.1074/jbc.M401656200

[R27] Mali P, Yang L, Esvelt KM, Aach J, Guell M, DiCarlo JE, Norville JE, and Church GM (2013). RNA-guided human genome engineering via Cas9. Science 339, 823–826. 23287722 10.1126/science.1232033 PMC3712628

[R28] McKenna A, Hanna M, Banks E, Sivachenko A, Cibulskis K, Kernytsky A, Garimella K, Altshuler D, Gabriel S, Daly M, and DePristo MA (2010). The Genome Analysis Toolkit: a MapReduce framework for analyzing next-generation DNA sequencing data. Genome Res. 20, 1297–1303. 20644199 10.1101/gr.107524.110 PMC2928508

[R29] Menon T, Firth AL, Scripture-Adams DD, Galic Z, Qualls SJ, Gilmore WB, Ke E, Singer O, Anderson LS, Bornzin AR, Alexander IE, Zack JA, and Verma IM (2015). Lymphoid regeneration from gene-corrected SCID-X1 subject-derived iPSCs. Cell Stem Cell 16, 367–372. 25772073 10.1016/j.stem.2015.02.005 PMC4545662

[R30] Nosaka T, van Deursen JM, Tripp RA, Thierfelder WE, Witthuhn BA, McMickle AP, Doherty PC, Grosveld GC, and Ihle JN (1995). Defective lymphoid development in mice lacking Jak3. Science 270, 800–802. 7481769 10.1126/science.270.5237.800

[R31] Notarangelo LD, Mella P, Jones A, de Saint Basile G, Savoldi G, Cranston T, Vihinen M, and Schumacher RF (2001). Mutations in severe combined immune deficiency (SCID) due to JAK3 deficiency. Hum. Mutat. 18, 255–263. 11668610 10.1002/humu.1188

[R32] Ohnuki MTK, and Yamanaka S (2009). Generation and characterization of human induced pluripotent stem cells. Curr. Protoc. Stem Cell Biol. Chapter 4, Unit 4A 2. 10.1002/9780470151808.sc04a02s9 19536759

[R33] Pai SY, Logan BR, Griffith LM, Buckley RH, Parrott RE, Dvorak CC, Kapoor N, Hanson IC, Filipovich AH, Jyonouchi S, (2014). Transplantation outcomes for severe combined immunodeficiency, 2000–2009. N. Engl. J. Med. 371, 434–446. 25075835 10.1056/NEJMoa1401177 PMC4183064

[R34] Park JH, Adoro S, Guinter T, Erman B, Alag AS, Catalfamo M, Kimura MY, Cui Y, Lucas PJ, Gress RE, (2010). Signaling by intrathymic cytokines, not T cell antigen receptors, specifies CD8 lineage choice and promotes the differentiation of cytotoxic-lineage T cells. Nat. Immunol. 11, 257–264. 20118929 10.1038/ni.1840 PMC3555225

[R35] Quinlan AR, and Hall IM (2010). BEDTools: a flexible suite of utilities for comparing genomic features. Bioinformatics 26, 841–842. 20110278 10.1093/bioinformatics/btq033 PMC2832824

[R36] Rausch T, Zichner T, Schlattl A, Stutz AM, Benes V, and Korbel JO (2012). DELLY: structural variant discovery by integrated paired-end and split-read analysis. Bioinformatics 28, i333–i339. 22962449 10.1093/bioinformatics/bts378 PMC3436805

[R37] Rice P, Longden I, and Bleasby A (2000). EMBOSS: the European Molecular Biology Open Software Suite. Trends Genet. 16, 276–277. 10827456 10.1016/s0168-9525(00)02024-2

[R38] Rothenberg EV, Moore JE, and Yui MA (2008). Launching the T-cell-lineage developmental programme. Nat. Rev. Immunol. 8, 9–21. 18097446 10.1038/nri2232 PMC3131407

[R39] Russell SM, Tayebi N, Nakajima H, Riedy MC, Roberts JL, Aman MJ, Migone TS, Noguchi M, Markert ML, Buckley RH, (1995). Mutation of Jak3 in a patient with SCID: essential role of Jak3 in lymphoid development. Science 270, 797–800. 7481768 10.1126/science.270.5237.797

[R40] Sauer AV, Di Lorenzo B, Carriglio N, and Aiuti A (2014). Progress in gene therapy for primary immunodeficiencies using lentiviral vectors. Curr. Opin. Allergy Clin. Immunol. 14, 527–534. 25207699 10.1097/ACI.0000000000000114

[R41] Schmitt TM, and Zúñiga-Pflücker JC (2002). Induction of T cell development from hematopoietic progenitor cells by delta-like-1 in vitro. Immunity 17, 749–756. 12479821 10.1016/s1074-7613(02)00474-0

[R42] Schmitt TM, de Pooter RF, Gronski MA, Cho SK, Ohashi PS, and Zúñiga-Pflücker JC (2004). Induction of T cell development and establishment of T cell competence from embryonic stem cells differentiated in vitro. Nat. Immunol. 5, 410–417. 15034575 10.1038/ni1055

[R43] Siepel A, Bejerano G, Pedersen JS, Hinrichs AS, Hou M, Rosenbloom K, Clawson H, Spieth J, Hillier LW, Richards S, (2005). Evolutionarily conserved elements in vertebrate, insect, worm, and yeast genomes. Genome Res. 15, 1034–1050. 16024819 10.1101/gr.3715005 PMC1182216

[R44] Smith C, Gore A, Yan W, Abalde-Atristain L, Li Z, He C, Wang Y, Brodsky RA, Zhang K, Cheng L, and Ye Z (2014). Whole-genome sequencing analysis reveals high specificity of CRISPR/Cas9 and TALEN-based genome editing in human iPSCs. Cell Stem Cell 15, 12–13. 24996165 10.1016/j.stem.2014.06.011 PMC4338993

[R45] Stenson PD, Ball EV, Mort M, Phillips AD, Shiel JA, Thomas NS, Abeysinghe S, Krawczak M, and Cooper DN (2003). Human Gene Mutation Database (HGMD): 2003 update. Hum. Mutat. 21, 577–581. 12754702 10.1002/humu.10212

[R46] Takahashi K, and Yamanaka S (2006). Induction of pluripotent stem cells from mouse embryonic and adult fibroblast cultures by defined factors. Cell 126, 663–676. 16904174 10.1016/j.cell.2006.07.024

[R47] Takahashi K, Tanabe K, Ohnuki M, Narita M, Ichisaka T, Tomoda K, and Yamanaka S (2007). Induction of pluripotent stem cells from adult human fibroblasts by defined factors. Cell 131, 861–872. 18035408 10.1016/j.cell.2007.11.019

[R48] Thomis DC, Gurniak CB, Tivol E, Sharpe AH, and Berg LJ (1995). Defects in B lymphocyte maturation and T lymphocyte activation in mice lacking Jak3. Science 270, 794–797. 7481767 10.1126/science.270.5237.794

[R49] Timmermans F, Velghe I, Vanwalleghem L, De Smedt M, Van Coppernolle S, Taghon T, Moore HD, Leclercq G, Langerak AW, Kerre T, (2009). Generation of T cells from human embryonic stem cell-derived hematopoietic zones. J. Immunol. 182, 6879–6888. 19454684 10.4049/jimmunol.0803670

[R50] Veres A, Gosis BS, Ding Q, Collins R, Ragavendran A, Brand H, Erdin S, Cowan CA, Talkowski ME, and Musunuru K (2014). Low incidence of off-target mutations in individual CRISPR-Cas9 and TALEN targeted human stem cell clones detected by whole-genome sequencing. Cell Stem Cell 15, 27–30, Erratum in Cell Stem Cell. 2014 Aug 7;15(2):254. Cowan, Chad A [added]. 24996167 10.1016/j.stem.2014.04.020 PMC4082799

[R51] von Freeden-Jeffry U, Solvason N, Howard M, and Murray R (1997). The earliest T lineage-committed cells depend on IL-7 for Bcl-2 expression and normal cell cycle progression. Immunity 7, 147–154. 9252127 10.1016/s1074-7613(00)80517-8

[R52] Wang K, Li M, and Hakonarson H (2010). ANNOVAR: functional annotation of genetic variants from high-throughput sequencing data. Nucleic Acids Res. 38, e164. 10.1093/nar/gkq603. 20601685 PMC2938201

[R53] Welter D, MacArthur J, Morales J, Burdett T, Hall P, Junkins H, Klemm A, Flicek P, Manolio T, Hindorff L, and Parkinson H (2014). The NHGRI GWAS Catalog, a curated resource of SNP-trait associations. Nucleic Acids Res. 42, D1001–D1006. 24316577 10.1093/nar/gkt1229 PMC3965119

[R54] Wen R, Wang D, McKay C, Bunting KD, Marine JC, Vanin EF, Zambetti GP, Korsmeyer SJ, Ihle JN, and Cleveland JL (2001). Jak3 selectively regulates Bax and Bcl-2 expression to promote T-cell development. Mol. Cell. Biol. 21, 678–689. 11134353 10.1128/MCB.21.2.678-689.2001 PMC86650

[R55] Ye K, Schulz MH, Long Q, Apweiler R, and Ning Z (2009). Pindel: a pattern growth approach to detect break points of large deletions and medium sized insertions from paired-end short reads. Bioinformatics 25, 2865–2871. 19561018 10.1093/bioinformatics/btp394 PMC2781750

[R56] Yu J, Vodyanik MA, Smuga-Otto K, Antosiewicz-Bourget J, Frane JL, Tian S, Nie J, Jonsdottir GA, Ruotti V, Stewart R, (2007). Induced pluripotent stem cell lines derived from human somatic cells. Science 318, 1917–1920. 18029452 10.1126/science.1151526

