# Supplementary material for: Modeling Human Severe Combined Immunodeficiency and Correction by CRISPR/Cas9-Enhanced Gene Targeting
Source: Cell Rep. Author manuscript; Available in PMC 2026 Apr 22. (PMC13101568; doi:10.1016/j.celrep.2015.08.013)
Supplement: Data Supplement [file NIHMS2156987-supplement-Data_Supplement.pdf]

Cell Reports

Supplemental Information

Modeling Human Severe Combined Immunodeficiency and Correction by CRISPR/Cas9-Enhanced Gene Targeting

Chia-Wei Chang, Yi-Shin Lai, Erik Westin, Alireza Khodadadi-Jamayran, Kevin M. Pawlik, Lawrence S. Lamb, Jr., Fred Goldman, and Tim M. Townes

Supplementary Figure 1

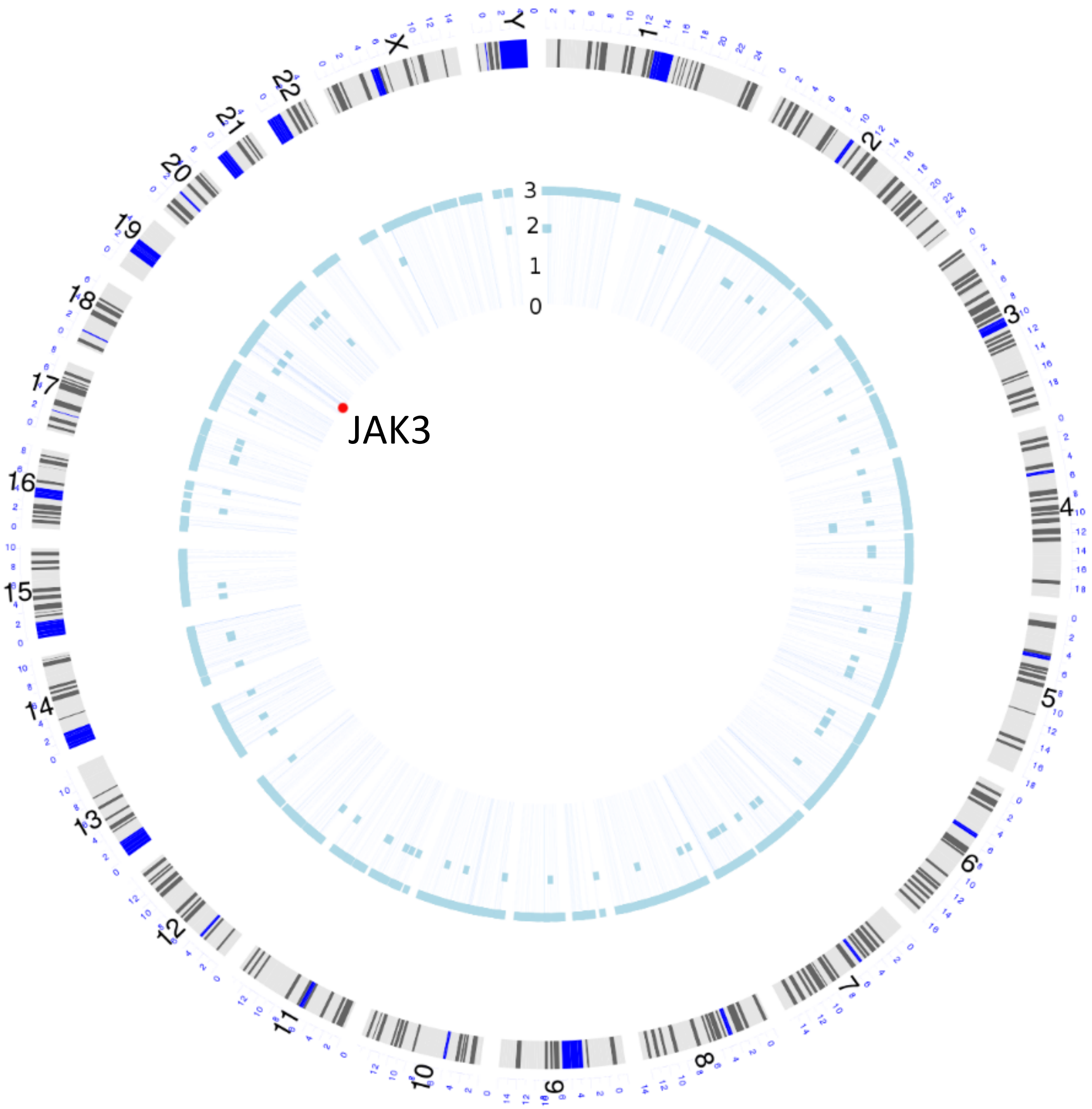

Genome distribution of potential off-target sites (See Whole Genome Sequencing and Analysis in Experimental Procedures and Table 1). Potential off-target sites were identified by aligning the CRISPR/Cas9 guide sequences to the hg19 reference genome using EMBOSS fuzznuc software (v6.6.0.0) (Rice et al., 2000) and allowing for a maximum of three mismatches; 1193 potential off-target sites were identified for the first guide sequence (GTGAGATACAGATACAGACA) and 257 sites were identified for the second guide sequence (AATGATTTGCCTGGAATGCC). Potential off-target sites are displayed using OmiCircos software (http://bioconductor.org/packages/release/bioc/html/OmicCircos.html).

Supplementary Figure 2

A.

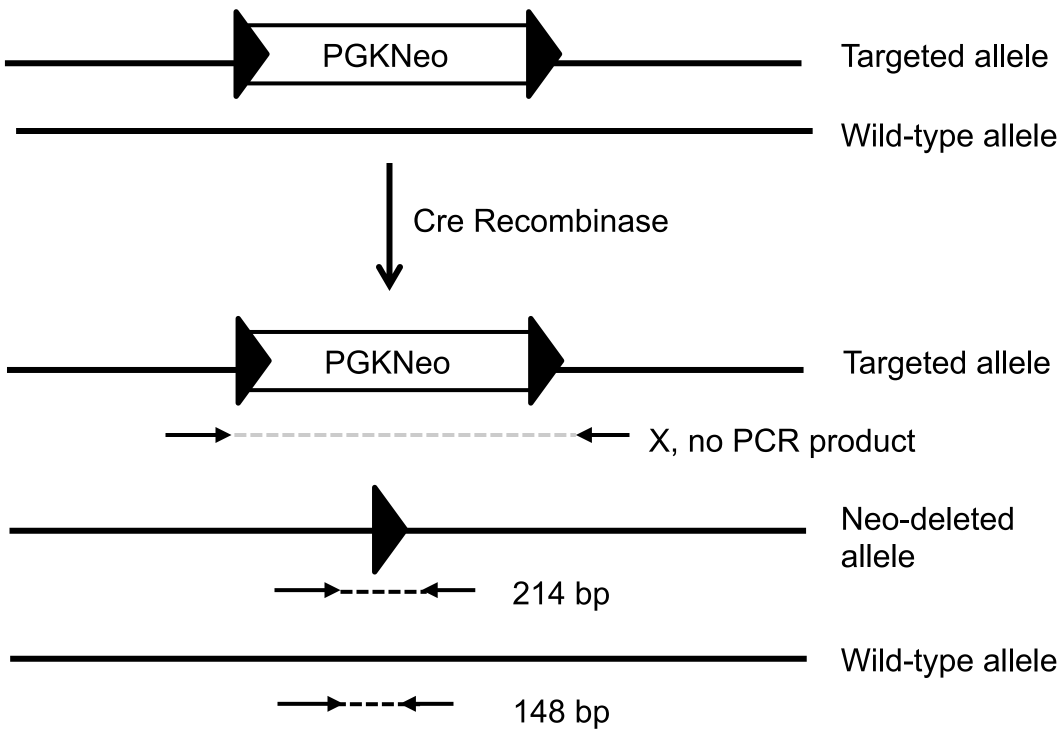

B.

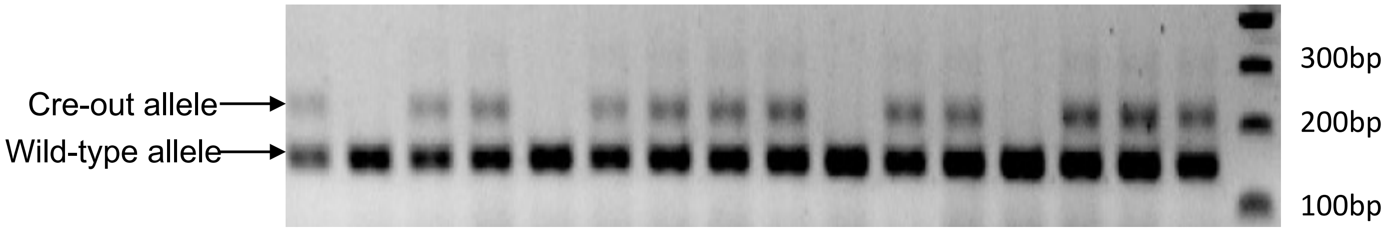

A. Strategy of genotyping iPSC clones after Cre-recombinase mediated selection marker excision (See Human iPSC reprogramming and characterization in Experimental Procedures). Triangles, loxP sites. Arrows, PCR primers.

B. A representative picture of PCR analysis. A heterozygous corrected iPSC line was infected with the Cre-expressing adenovirus (rAd-Cre-IE). The colonies were picked 2 weeks later and expanded for genomic DNA extraction. Each lane indicates individual iPSC clones. Amplicons derived from wild-type and Cre-out allele are 148 bp and 214 bp, respectively. This PCR condition (short extension time) can not generate amplicons from the allele that the selection marker still retained.
